# 3DAirSig: A Framework for Enabling In-Air Signatures Using a Multi-Modal Depth Sensor

**DOI:** 10.3390/s18113872

**Published:** 2018-11-10

**Authors:** Jameel Malik, Ahmed Elhayek, Sheraz Ahmed, Faisal Shafait, Muhammad Imran Malik, Didier Stricker

**Affiliations:** 1 German Research Center for Artificial Intelligence, DFKI, Kaiserslautern 67653, Germany; jameel.malik@dfki.de (J.M.); Didier.Stricker@dfki.de (D.S.); 2 Department of Informatics, University of Kaiserslautern, Kaiserslautern 67653, Germany; 3 School of Electrical Engineering and Computer Science (SEECS), National University of Sciences and Technology (NUST), Islamabad 44000, Pakistan; faisal.shafait@seecs.edu.pk; 4 University of Prince Mugrin (UPM), Madinah 20012, Saudi Arabia; ahmed.elhayek@dfki.de; 5 Deep Learning Laboratory, National Center of Artificial Intelligence (NCAI), Islamabad 44000, Pakistan

**Keywords:** in-air signature, depth sensor, convolutional neural network (CNN), 3D hand pose estimation, multidimensional dynamic time warping (MD-DTW)

## Abstract

In-air signature is a new modality which is essential for user authentication and access control in noncontact mode and has been actively studied in recent years. However, it has been treated as a conventional online signature, which is essentially a 2D spatial representation. Notably, this modality bears a lot more potential due to an important hidden depth feature. Existing methods for in-air signature verification neither capture this unique depth feature explicitly nor fully explore its potential in verification. Moreover, these methods are based on heuristic approaches for fingertip or hand palm center detection, which are not feasible in practice. Inspired by the great progress in deep-learning-based hand pose estimation, we propose a real-time in-air signature acquisition method which estimates hand joint positions in 3D using a single depth image. The predicted 3D position of fingertip is recorded for each frame. We present four different implementations of a verification module, which are based on the extracted depth and spatial features. An ablation study was performed to explore the impact of the depth feature in particular. For matching, we employed the most commonly used multidimensional dynamic time warping (MD-DTW) algorithm. We created a new database which contains 600 signatures recorded from 15 different subjects. Extensive evaluations were performed on our database. Our method, called 3DAirSig, achieved an equal error rate (EER) of 0.46%. Experiments showed that depth itself is an important feature, which is sufficient for in-air signature verification.

## 1. Introduction

Electronic identity authentication plays a vital role for access control and security in modern age. In e-authentication, a protected token (e.g., a cryptographic key) is used to access a system or an application on a network. Biometric-based authentication uses physical, behavioral, or adhered human characteristics for identification. These characteristics include, for instance, a fingerprint, iris scan, handwritten signature, color, gait, and facial scan. Biometric authentication is more secure and less prone to identity theft [1]. With the rapid growth of technology, emerging concepts, such as *classroom of the future* http://iql-lab.de [2], would allow smart interactions in a virtual and augmented reality environment. In such a noncontact mode of interaction, biometric in-air signature verification is important for access control and authentication. Traditionally, signature verification methods are classified into two types, namely, offline and online signature verification. In offline signature verification, a handwritten signature is acquired on a document and verified using a scanned or camera-captured image of the 2D signature [3,4,5]. The artificial neural network (ANN), support vector machine (SVM), and pixel matching technique (PMT) are famous classification algorithms, which have been used by offline methods. On the other hand, in online methods, e-signatures are taken on a touch device (e.g., tablet or pad) using an e-pen or finger movement on a digital screen [6,7,8,9,10,11,12,13]. These methods are difficult to forge due to various dynamic features, such as velocity, acceleration, and pen pressure. The signature acquisition techniques mentioned above exploit the 2D spatial and temporal information taken from a digital touch screen or a document. For verification, dynamic time warping (DTW) is the most effective and widely used technique [14,15], mainly because of its ability to well align temporal signals. Other prominent approaches based on a. neural network (NN) [12], SVM [13], and the hidden Markov model (HMM) [9] have also been employed for online verification.

In-air signatures are a new modality which allows a user to sign in the air by making free hand movements, thereby eliminating the need for a writing surface. Notably, this modality inherently contains important information in the third dimension (i.e., depth), in addition to the 2D spatial pattern. Existing methods for in-air signature verification use either an RGB or depth camera, a wearable camera (e.g., Google Glass) or a movement sensor in a cell phone [1,16,17,18]. However, these methods address the problem of in-air signature acquisition and verification in the conventional way. More precisely, the focus of these approaches has been inclined towards the utilization of the 2D spatial and temporal features. Lack of consideration towards the hidden depth information has restricted the exploration of the full potential in the 3D signature trajectory. In this work, we investigate the potential of the unique depth pattern. We show that the depth itself is a strong feature, which is sufficient for in-air signature verification. On the other hand, fingertip tracking is a challenging problem, especially due to the occlusions of fingers and viewpoint changes during signing freely in the air. The acquisition of a correct in-air signature trajectory is crucial to verification. This problem has not been well-addressed because the existing approaches try to locate only the fingertip using heuristics. Some of the approaches rely on palm center point tracking [17,19] which does not accurately mimic the pointing finger movement while signing in the air. Furthermore, due to their complex in-air signature acquisition systems, they are not suitable for real-time applications. In principle, the skeleton of a human hand is a kinematic structure where each child joint is connected to its parent joints [20,21]. Therefore, for a stable and reliable tracking of the position of a fingertip, the complete 3D pose of a hand should be estimated. In contrast to existing fingertip-tracking approaches, we exploited the huge progress of the convolutional-neural-network (CNN) based hand pose estimation using a low cost multimodal depth sensor [22] and trained a CNN to estimate the hand joints’ keypoints in 3D; see Section 4.3. Estimating a full hand pose is more stable, especially in the case of occluded fingertips, as it learns to estimate all features of the hand. We created our own database of in-air signatures for analysis and verification. We performed a detailed ablation study, which especially reveals the significance of the hidden depth feature in verification. We propose an improved spatial-features-based verification strategy which incorporates the depth information; see Section 6.1. We employed the most common and effective multidimensional dynamic time warping (MD-DTW) algorithm for matching, since our focus is to investigate and highlight the potential in individual features of the in-air signature using the best practice for verification.

## 2. Related Work

Comprehensive reviews on offline and online signature verification have been reported in References [23,24,25]. Keeping in view the relevance with our work, here we discuss the published literature on in-air signature verification. Katagiri et al. [26] proposed the first free space personal authentication system. They adopted a high-speed video camera to acquire an in-air signature trajectory. For verification, they employed a commercial signature verification engine provided by CyberSIGN Japan Inc. (Tokyo, Japan) http://www.cybersign.com. In Reference [27], Takeuchi et al. combined hand shape features with an RGB camera to capture handwriting motion in the air. Keeping in view the extended use of smartphones in various applications, Diep et al. [28] used a motion sensor in a smartphone to record signature data. They used SVM for verification. Matsuo et al. [29] introduced an adaptive template update method in order to improve long-term stability in arm-swing motion. Jeon et al. [17] adapted a low-cost depth camera to capture an in-air signature trajectory. In order to record the signature trajectory, they introduced a heuristic approach to detect the palm center position. Bailador et al. [18] investigated various pattern recognition techniques, i.e., HMM, Bayes classifier, and DTW, for authentication. The best performance was shown by the DTW algorithm. In order to capture in-air signature trajectory, the authors used an embedded 3D accelerometer in a mobile phone. With the recent trend towards wearable technology, Sajid et al. [1] proposed a new in-air signature acquisition method using Google Glass. They used a motion-based video segmentation algorithm along with a skin-color-based hand segmentation in order to acquire signature data. A video-based in-air signature verification system using a high-speed RGB camera was introduced by Fang et al. [16]. They traced the fingertip using an improved tracking learning detection (TLD) algorithm. For the verification phase, the authors developed a fusion algorithm based on an improved DTW and the fast Fourier transform (FFT). Recently, Khoh et al. [19] proposed a predictive palm segmentation algorithm to create a motion history image (MHI) using a depth sensor. Afterwards, they produced a two-dimensional representation of a hand-gesture signature based on the MHI. All of the methods mentioned above treat and process in-air signature trajectories in the conventional online form. However, we emphasize that in-air signatures enclose a unique hidden depth feature, which should not be ignored in acquisition and verification. In this work, we investigate the potential of this important feature. On the other hand, the reported methods for fingertip tracking are based on heuristics, which are not feasible for practical applications. Inspired by the recent progress in deep-learning-based hand pose estimation using a depth sensor [22], we propose a new real-time algorithm for in-air acquisition which regresses the 3D hand pose rather than detecting only the fingertip or palm center. Therefore, the proposed method is not restricted to any specific hand pose and has the ability to perform well in cases of occlusion.

## 3. Framework Overview

**Figure 1:**
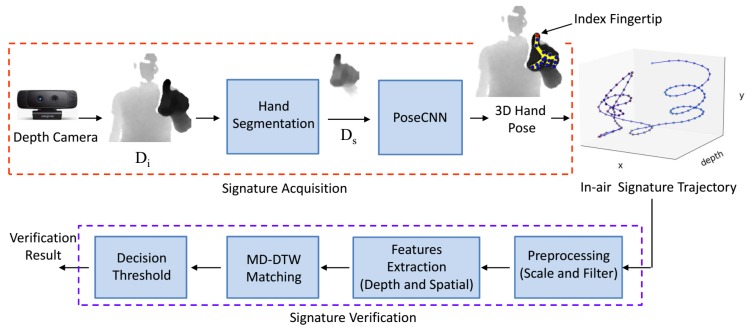
An overview of our method for in-air signature acquisition and verification. In the acquisition phase, the hand region is first segmented from a raw depth frame. Then, the estimated 3D position of the index fingertip is recorded for every frame using a CNN-based hand pose estimation method. For verification, the test signature is scaled and filtered. Thereafter, the spatial and depth features are extracted for matching using the MD-DTW algorithm. Finally, the test signature is verified by the decision threshold.

The block diagram of our proposed 3D in-air signature acquisition and verification framework is shown in Figure 1. For the signature acquisition, we propose a CNN-based hand pose estimation method to predict the 3D hand joint positions from a single depth image. The input depth frame $D_{i}$ is captured using Intel’s creative senz3D depth camera [30]; see Section 4.1 for details of our acquisition setup. The hand region is segmented from $D_{i}$ using center of hand mass (CoM) followed by a crop function; see Section 4.2. The output $D_{s}$ is fed to the PoseCNN, which predicts the 3D hand pose; see Section 4.3. The estimated joint position of the index fingertip in each depth frame is used to record the 3D signature trajectory. The recorded in-air signature trajectory is preprocessed for normalization and smoothing; see Section 5.1. Thereafter, spatial and depth features are extracted from the 3D signature. For matching, MD-DTW is used to obtain a similarity measure between the selected feature of the preprocessed test signature and the corresponding precomputed feature template. In the final step, the test signature is verified by the decision threshold; see Section 5.3 and Section 5.4.

## 4. In-Air Signature Acquisition

In this section, we explain our 3D in-air signature acquisition setup, fingertip-tracking approach, and the dataset creation.

### 4.1. Data Acquisition Setup

**Figure 2:**
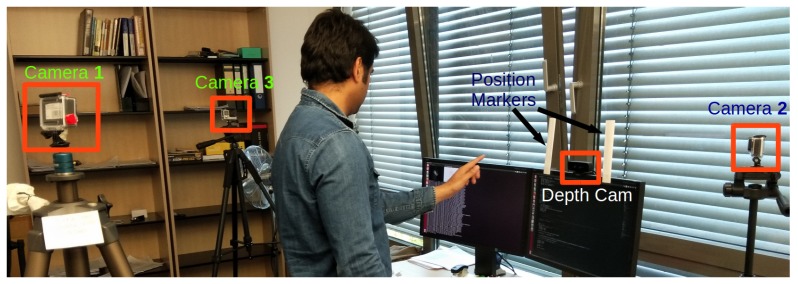
Our setup for in-air signature acquisition. The depth camera is mounted on top of the screen. The position markers on both sides of the depth camera allow capturing of in-air signature within the field of view (FoV) of the camera. Three GoPro cameras are placed around a user to record the hand motion in 3D space from different view points. Camera 3 specifically records the depth variation.

Figure 2 shows our in-air signature acquisition setup. A user is allowed to sign freely in the air within the field of view (FoV) of Intel’s creative senz3D depth camera mounted on top of the screen. The FoV of the camera is $74^{\circ }$ diagonal. Two position markers are placed on either side of the depth camera to provide an approximate start and end position for recording the signature. Our acquisition system allows to easily select between left or right hand before signing. During the signature acquisition, the user’s hand should be the closest object to the camera. Notably, our method is not restricted to a specific hand pose for signing in the air. However, most of the users participating in our database creation used a natural pointing index finger pose (as shown in Figure 1). Our system allows a user to see a 2D projection of the 3D signature trajectory in real-time on a signature pad, which is displayed on a monitor screen. Our acquisition system is robust to variations in ambient light intensity in indoor environments.

### 4.2. Hand Segmentation

**Figure 3:**
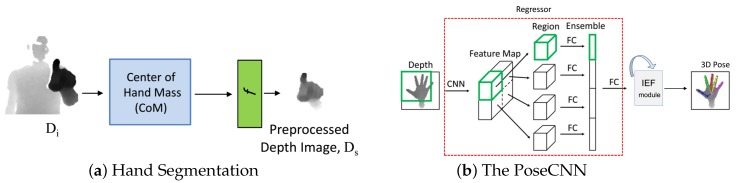
(**a**) shows our approach for hand segmentation from a raw depth frame. First, the center of hand mass (CoM) is calculated, provided that the hand is the closest object to the depth camera. Then, the function *f* crops the hand region in 3D. (**b**) The PoseCNN takes the cropped hand image as input and regresses 3D sparse joints keypoints.

An accurate segmentation of the hand region from a raw depth frame is important for learning-based hand pose estimation approaches. We used a hand segmentation method similar to that described in Reference [31] (Figure 3a). The segmentation process has two steps. The first step is to find an accurate 3D location of the hand palm center. As mentioned earlier, the hand is assumed to be the closest object to the camera; therefore, a simple depth value-based thresholding can be used to separate the human body from the hand. We used a depth threshold of 600 mm. Then, the 3D location of the palm center is calculated by averaging all the pixels which belong to the hand region (i.e., pixel values less than 600 mm). The second step is to preprocess or crop the hand region in 3D using the obtained palm center. In Figure 3a, the function *f* crops the hand region around the calculated palm center using a bounding box. The size of the bounding box is 150 mm. Then, depth values are normalized to [−1,1]. The resultant image is of a size of 96 × 96. The runtime of our hand segmentation method is 0.47 ms.

### 4.3. Fingertip Tracking

Stable and reliable fingertip tracking is essential for the correct recording of a 3D in-air signature. For this purpose, we exploited the huge progress of CNN-based hand pose estimation methods. One of the major advantages associated with these methods is that they estimate the complete hand pose rather than detecting only the fingertip or palm center. This is particularly important in cases of severe occlusions of fingers during signing in the air. An overview of our method is shown in Figure 3b. The PoseCNN is used to estimate the 16 3D joint positions of the hand skeleton from a single depth image. The first part of the PoseCNN (i.e., Regressor) is adopted from [31], which originally regressed 3D hand poses using a single shared CNN for feature extraction and a powerful yet simple region ensemble (REN) strategy. In our implementation, the final fully connected (FC) layer of the regressor outputs features $\varphi \in R^{512}$ instead of joint positions.

*Architecture of the Regressor*: The architecture of the shared CNN for feature extraction comprises six convolution layers using 3 × 3 kernel sizes. A rectified Linear Unit (ReLu) is connected with each of the convolution layers as an activation function. A max pooling layer with a stride of 2 is connected after every consecutive pair of convolution layers. Two residual connections are incorporated between the pooling layers. The output features are of size 12 × 12 × 64. Then, two FC layers of dimension 2048 are connected with a dropout ratio of 0.5. As shown in Figure 3b, the feature maps from different regions of the input depth image are divided into a 2 × 2 grid. Thereafter, the features from the FC layers of the grid regions are simply concatenated. The final FC layer after the concatenation produces $\varphi \in R^{512}$. We refer the reader to Reference [31] for further details of the shared CNN architecture and the REN strategy.

*IEF module*: We integrate an iterative error feedback (IEF) module to the end of the regressor for refinement of the estimated hand pose. The output of the regressor φ is concatenated with an initial estimate of hand pose $H_{p}$ i.e., $\phi =\{\varphi ,H_{p}\}$. $H_{p}$ is obtained by averaging all the joint positions from the ground truth annotations of the datasets. ϕ is fed to the IEF module, which comprises two FC layers with 512 neurons each. Both the FC layers use dropout layers with a ratio of 0.3. The last FC layer contains 48 neurons, corresponding to the 16 3D joint positions. The IEF module basically refines $H_{p}$ in an iterative feedback manner such that $H_{p}\left(t+1\right)=H_{p}\left(t\right)+\delta H_{p}\left(t\right)$. We use three iterations.

*Training of the PoseCNN*: In order to improve the generic performance of the PoseCNN, especially for varying hand shapes, we trained on a combined dataset (i.e., *HandSet*) proposed in Reference [21]. The *HandSet* encapsulates three famous public hand pose datasets in a single unified format. These datasets include NYU [32], ICVL [33], and MSRA-2015 [34]. Our network runs on a desktop using Nvidia’s Geforce GTX 1080 Ti GPU. We used a learning rate (LR) of 0.001 with a 0.9 stochastic gradient descent (SGD) momentum and a batch size of 256. One forward pass through the PoseCNN takes 3.2 ms.

*Accuracy of predicted fingertips positions*: We quantitatively evaluated the accuracy of estimated fingertips positions on the NYU test dataset. The 3D joint location error on fingertips comes out to be 13.2 mm, which is better than the lowest reported error (15.6 mm) in Reference [35]; see Table 1.

### 4.4. The Dataset Creation

**Figure 4:**
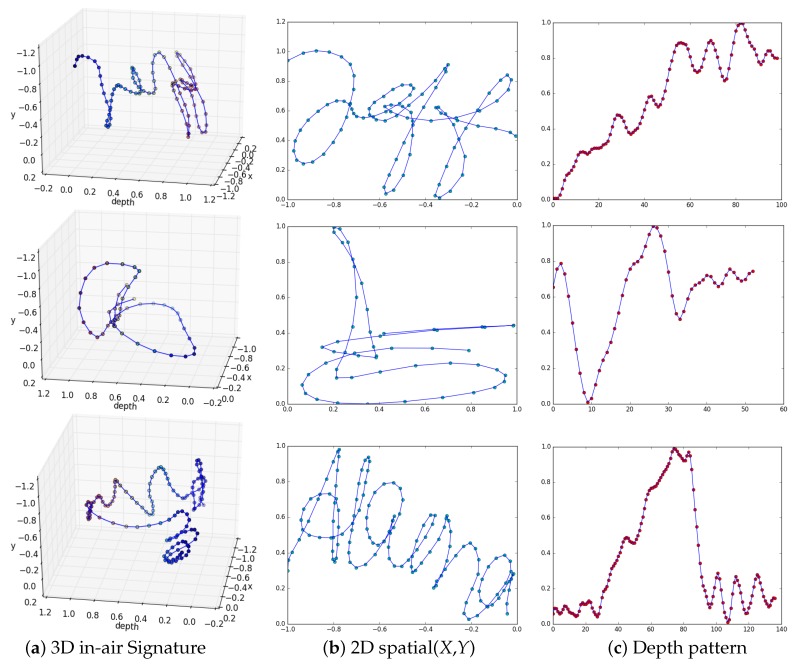
Samples of genuine in-air signatures from our dataset. Each one of the rows shows (**a**) the 3D in-air signature trajectory, (**b**) the 2D spatial view, and (**c**) depth pattern. The depth pattern of each signature is particularly unique and, therefore, it is an important hidden feature.

There are two main motivations for creating our dataset for in-air signature verification. The first is to study the potential of the hidden depth feature. The second is to exploit the great progress in CNN-based hand pose estimation for stable and reliable fingertip tracking. For video recordings of genuine signatures which are shown to impostors, we used three GoPro cameras in our capture setup; see Figure 2. Two of the cameras (Cameras 1 and 2) were placed behind and right-front of the subject to record the spatial pattern of the signature. The third camera (Camera 3) recorded from the side view to visualize the depth variation in the signature. The users were asked to practice multiple times before the actual recordings as signing in the air is generally not a well-familiar modality. We emphasized on making explicit variations in depth during signing, which allows to fully exploit the hidden depth feature in the in-air signature trajectory. Our database (the dataset will be publicly available at https://goo.gl/yFdfdL) includes 600 signatures from 15 users. We recorded 15 genuine signatures from each of the users and obtained 25 forgeries for every original writer from 5 impostors. Ten out of 15 genuine signatures were used for the testing phase and the remaining were used for the training phase; see Section 5. Samples of genuine preprocessed signatures with the corresponding 2D spatial views and unique depth patterns are shown in Figure 4. The color variations in the 3D view of a signature show variation in the depth pattern; see Figure 4a. Notably, each signature has a unique depth pattern (Figure 4c) which is challenging to forge jointly with the spatial pattern; see Section 6.

## 5. In-air Signature Verification

In this section, we explain the preprocessing, extracted features, training, and testing phases. We adopted a commonly used MD-DTW algorithm for matching, mainly because it can align temporal signals well even though they are not consistent in time.

### 5.1. Preprocessing

**Figure 5:**
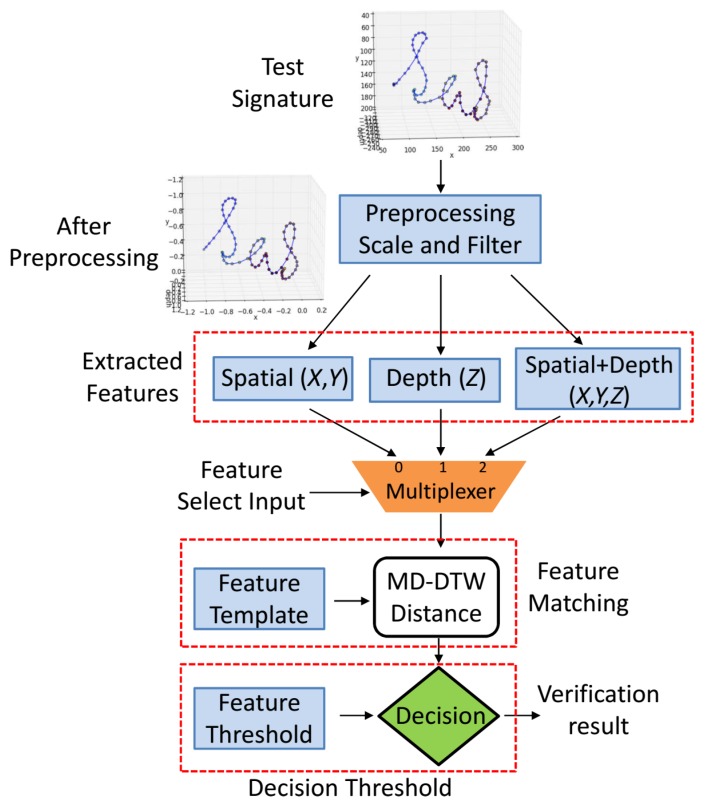
The flow diagram of the testing phase of our in-air signature verification system. The test signature is preprocessed for normalization and smoothing. The extracted features include spatial, depth, and spatial plus depth. Then, a multiplexer with a control input is used to select one of the extracted features. The selected feature is matched with the corresponding feature template using the MD-DTW algorithm. Finally, the verification result is produced by the decision threshold.

The recorded in-air signature is preprocessed for normalization and smoothing. An appropriate preprocessing of a signature can affect the results of signature verification [11,17]. First, we removed a few redundant 3D points from the start and end of a signature trajectory whose displacement was less than 3 pixels. The removed points corresponded to a small wait time before starting the actual hand motion and a time to close the recording after the end of the signature. In order to remove discontinuities due to fast hand movements, we applied a moving average filter with a window size of 5, which resulted in a smoother signature trajectory. Thereafter, we normalized the signatures to compensate for variations in *position* and *scale*. For normalization, the transformation from absolute to relative values in 3D can be obtained using the following formulas:

(1) $$X_{j}^{*}=\left(X_{j}-X_{min}\right)/\left(X_{max}-X_{min}\right)$$

(2) $$Y_{j}^{*}=\left(Y_{j}-Y_{min}\right)/\left(Y_{max}-Y_{min}\right)$$

(3) $$Z_{j}^{*}=\left(Z_{j}-Z_{min}\right)/\left(Z_{max}-Z_{min}\right),$$

where $X_{j}$,$Y_{j}$, and $Z_{j}$ are the original or absolute values of a signature. $X_{j}^{*}$, $Y_{j}^{*}$, and $Z_{j}^{*}$ are the transformed values. $X_{min}$, $X_{max}$, $Y_{min}$, $Y_{max}$, $Z_{min}$, and $Z_{max}$ are the minimum and maximum values of $X_{j}$, $Y_{j}$, and $Z_{j}$. A test signature before and after the preprocessing step is shown in Figure 5.

### 5.2. Feature Extraction

**Figure 6:**
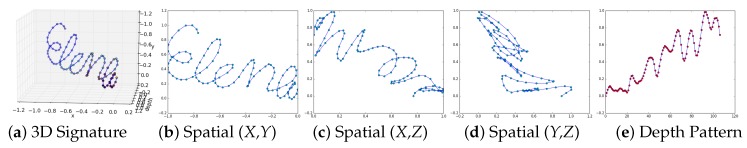
Illustration of different features which are used for in-air signature verification. We fully exploited different combinations of the features inherently present in the in-air signature trajectory to improve the performance of the verification system. The unique depth feature of a user especially plays a vital role in verification phase.

Figure 6 shows all the feature combinations we used in our verification process. We studied the impact of the hidden depth feature in different ways. The spatial (*X*,*Y*) is a commonly used 2D representation of in-air signatures; see Figure 6b. However, we argue that only the spatial (*X*,*Y*) is not a complete representative of an in-air signature trajectory. Therefore, we extracted two new types of spatial features, i.e., spatial (*X*,*Z*) and spatial (*Y*,*Z*) which implicitly incorporate the depth feature. We also studied the impact of these two features when combined with the spatial (*X*,*Y*); see Section 6. Nevertheless, the most interesting feature is the hidden depth pattern (Figure 6e) which has not been fully explored in the previous works.

### 5.3. Training Phase

In this phase, we computed the feature templates and the respective feature thresholds using 75 genuine training samples. We used neither forgeries nor original signatures from the test set. It is worth noting that many pattern recognition researchers use models, e.g., NN, SVM, while training them on the positive (genuine) and negative (forgery) samples at the same time [37,38]. According to forensic handwriting examiners [39], this is unrealistic as, in the real world, one can never limit the forgery set and every signature, other than the concerned genuine signatures, can be considered a forgery. Furthermore, in real forensic cases, a verification system can only have genuine specimen samples and one or more questioned signatures. Henceforth, the best approach while using such models is to train them only on genuine specimen signatures. This can be done using specialized one class classifiers, like SVM/NN, for one class classification [40,41,42,43]. As explained earlier, we used five features; see Figure 6. Hence, a total of five feature templates and five respective feature thresholds for each of the 15 users are computed. A feature template is generated by averaging the features of the five training samples. We calculated a feature threshold value from five training samples of a signee, which are reserved for the training phase using the 4-fold cross validation strategy (i.e., using limited signatures for estimating how the system will perform when used to make predictions on data not used during training.

*4-fold cross validation strategy*: In this methodology, we randomly shuffled five genuine training signature samples and divided them into two groups. The first group contained four training samples, which were taken as the training set. The second group contained only one training sample, which was considered the dummy test set. More specifically, let $S=\{S_{t_{1}},S_{t_{2}},S_{t_{3}},S_{t_{4}},S_{t_{5}}\}$ be the five training samples of a signature, where $S_{x}\in R^{dxL_{x}}$. $L_{x}$ is the length of the signal $S_{x}$ and *d* is the number of dimensions of one point in the signal. In the first round, we split S into two subsets, $S_{a}=\left\{S_{t_{2}},S_{t_{3}},S_{t_{4}},S_{t_{5}}\right\}$ and $S_{b}=\left\{S_{t_{1}}\right\}$. This is simply taking the first sample $S_{t_{1}}$ out of comparison in this round. For $S_{a}$, we make a 4 × 4 confusion matrix $C_{1}$ using Equations (4) and (5). From $C_{1}$, we manually select a threshold value $th_{1}$ such that any compared threshold value greater than $th_{1}$ will declare the signature as forged. In the second round, we eliminate $S_{t_{2}}$ and calculate another 4 × 4 matrix $C_{2}$ and find $th_{2}$. In a similar way, we calculate $C_{3}$, $C_{4}$, and $C_{5}$ and select the respective thresholds $th_{3}$, $th_{4}$, and $th_{5}$. Finally, we simply take the mean $th_{m}$ of these five threshold values. The $th_{m}$ is used in the final decision threshold process.

### 5.4. Testing Phase

Figure 5 shows the flow chart of the testing phase. After the preprocessing step and the feature extraction, a feature select input of a 3 × 1 multiplexer allows to select one of the features, i.e., spatial, depth, or spatial plus depth. After the selection of a desired feature, a similarity measure is found with the corresponding feature template using the MD-DTW algorithm [44] as follows:

*MD-DTW Matching*: Let $s_{1}\in R^{dxL_{s_{1}}}$ and $s_{2}\in R^{dxL_{s_{2}}}$ be the two time series signals, where $L_{s_{1}}$ and $L_{s_{2}}$ are the lengths of $s_{1}$ and $s_{2}$, respectively, and *d* is the dimension of a single point in the signal. The distance matrix M(i,j) can be computed using the L2-norm without square root operation as:

(4) $$M(i,j)=\sum_{k=1}^{d}{\left(s_{1}\left(k,i\right)-s_{2}\left(k,j\right)\right)}^{2}.$$

After obtaining the matrix M(i,j), the distance or similarity score between the elements of $s_{1}$ and $s_{2}$ on the DTW path can be found using the following equation:

(5) $$D(i,j)=M(i,j)+\min\left\{\begin{array}{c} \begin{array}{c} D(i-1,j)\\ D(i-1,j-1)\\ D(i,j-1) \end{array} \end{array}\right.$$

*Decision Threshold*: In the final step, as shown in Figure 5, the obtained similarity score is simply compared with the corresponding feature threshold $th_{m}$; see Section 5.3. The test signature is verified if the DTW distance is less than the feature threshold.

## 6. Experiments and Results

In this section, we detail the experiments performed on our dataset. The performances are reported using the false rejection rate (FRR), false acceptance rate (FAR), and equal error rate (EER) as evaluation metrics.

### 6.1. Ablation Study

In this subsection, we detail the ablation study, which was performed on the extracted features (Figure 6). The impact of every feature on the performance of verification was investigated and the results are reported on our captured dataset. We propose four different implementations of a verification module based on the extracted features from the in-air signature trajectory.

**Figure 7:**
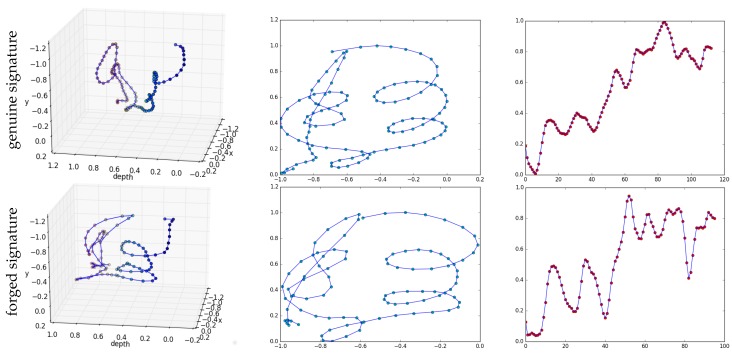
Comparison of spatial and depth patterns of the genuine and the corresponding forged signature. The top row shows a sample of a genuine signature and its corresponding spatial and depth patterns and the bottom one shows the respective forged signature. The color change shows the variation in depth pattern (3D view in the first column). Clearly, the depth pattern of the forged signature is different than the original one, although spatially they seem to be close.

*Depth-based signature verification (DSV) module*: To study the effectiveness of the hidden depth feature in verification, we implemented the verification module based on only the 1D depth *Z* of the signature trajectory. In Figure 5, the feature select input of the multiplexer is set to 1 in order to select the extracted depth feature from the test signature. The distance measure between the depth feature of the test signature and the precomputed depth feature template was calculated using Equations (4) and (5). The obtained similarity score was compared with the precomputed depth feature threshold to verify the test signature. Quantitative results on individual users are shown in Table 2. In Table 3, the *DSV* module shows FAR, FRR, and EER of 1.33%, 2.00%, and 0.51%, respectively. Qualitatively, the depth patterns of the genuine and forged signatures are shown in Figure 7. Despite the fact that the spatial patterns of the forgeries are closer to the genuine signatures, the depth patterns are distinct. As mentioned ealier, the impostors were shown the video recordings of the signatures from different camera views. However, they were either unable to notice exact variations in depth or it was difficult to forge the depth pattern. These results show the importance of the depth feature, which alone can provide a reliable verification. We also observed that it is more challenging for the impostor to forge the depth pattern simultaneously with the spatial pattern.

*2D spatial-based signature verification (SSV) module*: We implemented this verification module using only the 2D spatial (*X*,*Y*) feature; see Figure 6b. The feature select input of the multiplexer was set to 0; see Figure 5. The similarity score between the extracted spatial feature of the test signature and the spatial (*X*,*Y*) feature template was obtained using Equations (4) and (5). Then, the DTW distance was compared to the spatial feature threshold for the verification. Quantitative results are shown in Table 2 and Table 3. The performance of this verification module shows that considering only the spatial feature (*X*,*Y*) of the in-air signature trajectory results in a larger number of false acceptances and false rejections, thereby producing higher error rates.

**Figure 8:**
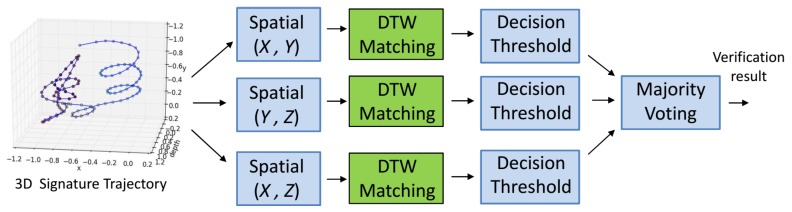
Our framework for an improved 2D spatial-based signature verification (*ISSV*) module. The spatial features (i.e., (*X,Y*), (*Y,Z*) and (*X,Z*)) are separately matched with the respective precomputed feature templates using a 2D-DTW algorithm. Thereafter, binary decisions are made by the decision thresholds. Lastly, the test signature is verified using a simple majority voting scheme.

*Improved 2D spatial-based signature verification (ISSV) module*: We attempted to improve the performance of the *SSV* module by incorporating additional spatial feature combinations (i.e., Spatial (*X*,*Z*) and Spatial (*Y*,*Z*)). The block diagram of the *ISSV* module is shown in Figure 8. The DTW matching is performed on these additional features in parallel to the traditional spatial (*X*,*Y*) using precomputed respective feature templates. Thereafter, binary decisions were obtained for each individual feature using the corresponding feature thresholds. Lastly, the final verification result was produced by a simple majority voting scheme, which declared the test signature as verified if no less than 2 features passed the corresponding decision thresholds. The verification results are reported in Table 2 and Table 3 that clearly show an improved performance compared to the *SSV* module. There is a notable reduction in the number of false acceptances and false rejections. The EER is reduced by 15.9% compared to the *SSV* module. However, the performance is still lagging behind the *DSV* module.

*3D signature verification (3D-SV) module*: In this verification module, we exploited the full 3D information (i.e., X,Y,Z) altogether. In Figure 5, the feature select input of the multiplexer was set to 2. The spatial plus depth feature (See Figure 6a) of the test signature was matched with the feature template and verified using the decision threshold. Quantitatively, Table 2 and Table 3 show that number of false rejections and FRR of this verification module are the same as those for the *DSV* module, whereas the number of false acceptances, FAR, and EER are reduced. In summary, Our *3D-SV* module shows the best performance, since it includes complete 3D information altogether, which is inherently present in the in-air signature trajectory.

### 6.2. Comparison with Other Verification Methods

Since there are no publicly available datasets and codes available for in-air signatures, Table 4 lists the performances of other methods evaluated on their self-built datasets. Alongside, we show the performance of our two best implementations on our self-built dataset. Our *DSV* module shows the competitive performance, whereas the *3D-SV* module shows the best results. It shows that the hidden depth feature in the in-air signature is important for improved performance.

## 7. Conclusions and Future Work

In this paper, we presented a real-time automatic in-air signature acquisition and verification framework using a low cost multi-modal depth camera. This paper addresses two major limitations in the existing methods for in-air signature verification. First, given the fact that the existing approaches use heuristic methods for fingertip tracking, which are unstable and impractical, we proposes a new CNN-based hand pose estimation method, which reliably tracks fingertips in real-time. The signature trajectory is recorded using an estimated 3D position of the index fingertip in each depth frame. Second, to explore the potential of the hidden depth feature in the in-air signature trajectory, we created our own dataset, which consists of 600 signatures recorded from 15 different subjects. We investigated the performance of the verification module by performing an ablation study on the spatial and depth features and performed extensive evaluations on our database. Experiments showed that the depth feature itself is sufficient for in-air signature verification. In the future, we plan to extend our database and develop a CNN-based algorithm for in-air signatures classification and matching.

## Figures and Tables

**Table 1:** The table shows the mean 3D joint location error (mm) for fingertips of various methods on the NYU [32] hand pose test dataset.

Method	Fingertips 3D Joint Location Error
DeepModel [20]	24.4 mm
Oberweger et al. [36]	23.2 mm
REN [35]	15.6 mm
**Ours**	13.2 mm

**Table 2:** The table shows the results of the four verification modules on our dataset. The number of false rejections (FR), false acceptances (FA), and total errors are provided for each of the 15 users. The *3D-SV* module shows the least number of FA, while its number of FR is equivalent to the *DSV* module.

Subject	1	2	3	4	5	6	7	8	9	10	11	12	13	14	15	Error Number
DSV																
FR	0	0	0	0	0	0	0	1	0	0	0	0	1	0	1	3
FA	0	1	0	0	0	0	0	0	0	0	1	0	1	2	0	5
SSV																
FR	0	1	0	0	0	0	0	1	0	0	0	1	2	0	3	8
FA	0	2	0	0	0	1	0	2	0	0	2	0	1	3	0	11
ISSV																
FR	0	1	0	0	0	0	0	0	0	0	0	1	1	0	2	5
FA	0	1	0	0	0	0	0	1	0	0	1	0	1	2	0	6
*3D-SV*																
FR	0	1	0	0	0	0	0	0	0	0	0	1	0	0	1	3
FA	0	0	0	0	0	0	0	0	0	0	1	0	0	0	0	3

**Table 3:** The table shows the person independent FAR, FRR, and EER for each of the four verification modules. There are a total of 150 genuine test and 375 forged signatures. The *3D-SV* module shows the best results, while the *DSV* module demonstrates competitive performance.

Verification Module	FAR (%)	FRR (%)	EER (%)
*DSV*	1.33	2.00	0.51
*SSV*	2.93	5.33	0.69
*ISSV*	1.60	3.34	0.58
*3D-SV*	0.80	2.00	0.46

**Table 4:** The table shows the performances of the existing in-air signature methods and our method. Due to unavailability of a public dataset for in-air signatures, we report results on our dataset. While our *3D-SV* module shows the best results, our *DSV* module, which is based on only depth analysis, shows the competitive performance.

Method	Dataset/Acquisition Method	Result
Nguyen et al. [28]	self-built/Accelerometer	EER: 0.8%
Hasan et al. [1]	self-built/Google glass	Accuracy = 97.5%
Nidal et al. [45]	self-built/ data glove	EER: 2.37%
Jeon et al. [17]	self-built/ depth camera	EER: 0.68%
Moon et al. [46]	self-built/Wifi signal	EER: 4.31%
Yuxun et al. [16]	self-built/RGB camera	FAR: 1.90% and FRR: 2.86%
*DSV*[**Ours**]	self-built/depth camera	EER: 0.51%
*3D-SV*[**Ours**]	self-built/depth camera	EER: 0.46%
